# Pick-and-Place Operation of Single Cell Using Optical and Electrical Measurements for Robust Manipulation

**DOI:** 10.3390/mi8120350

**Published:** 2017-11-30

**Authors:** Moeto Nagai, Keita Kato, Kiyotaka Oohara, Takayuki Shibata

**Affiliations:** Department of Mechanical Engineering, Toyohashi University of Technology, Toyohashi, Aichi 441-8580, Japan; bari91.ape50@gmail.com (K.K.); oohara.kiyotaka@gmail.com (K.O.); shibata@me.tut.ac.jp (T.S.)

**Keywords:** micro-manipulation, single cell, cell manipulation, electrical detection, cell screening, micro-well

## Abstract

A robust pick and placement operation of a single cell is necessary for efficient sample collection. Detection and manipulation of single cells requires minimum invasiveness. We report a less-invasive method for picking up and placing single cells using optical and electrical observations for robust cell manipulation. We measured the ionic current through a glass pipette during a cell capture and release operation to detect its capture. Trapping a cell on the pipette tip by suction decreased the current and allowed the detection of cell capture within 1 s. A time-series ionic current was sensitive to the location of a cell and effective at detecting a single cell. A time-series ionic current had a higher signal-to-noise ratio than time-series microscope images. Cell membrane integrity was analyzed at the different capturing and voltage conditions. Serum protein coating shows improvement of a cell release from a pipette tip. Measurement of trajectory and distance of a cell reveals that the movement depends on an ejection flow and the flow in a dish. We achieved a pick-up and placement operation for single cells that was compatible with an open-top microwell while performing observations using optical microscopy and measurements using an electrical current.

## 1. Introduction

A pick-up and placement operation for single cell targets is essential to find samples that exhibit a desired function [1,2,3]. Single-cell manipulation—the ability to place single cells in a target location with high viability—is a fundamental technique. A general concept of cell capturing and displacement consists of three steps, (1) cell measurement; (2) cell pick up; and (3) cell release. Screened cells are used for secretion and investigation of cell function. The application areas are regenerative medicine and in the production of biopharmaceuticals. A limitation of the current state-of-the-art is the lack of a robust and less invasive approach. Ideally, picking up and placing single cells in open-top microwells is required, which provides three advantages: (1) cells in open-top containers are accessible; (2) confining cells ensures that they remain in their positions; and (3) cells’ addresses are specified at each location. Another requirement is robust and less-invasive single cell manipulation. Robust manipulation can be established using a steady measurement method for a single cell.

Single cells have been patterned using various methods such as dielectrophoresis [4,5], microfluidics [6,7], optoelectronic tweezers [8,9,10,11], and surface acoustic waves [12,13]. These methods were used to place single cells at designed locations while optimizing their pattern. Optical tweezers have been used for displacing single cells into microwells [10,11]. However, in general it has not been sufficiently easy to screen single cell targets from a designated open-top microwell. The primary means of single-cell pickup is still manipulation with a pressure-control source and mechanical stages. A glass capillary has been used to pick up and place single cells [1,2,3,14,15,16]. Some systems are commercially available from companies that include Eppendorf and Narishige. These systems provide the desired functions, i.e., a high degree of manipulation freedom at a relatively low cost. Some researchers sucked a cell into the hole of a capillary tube and held it at the tip or inside a channel. In another case, instead of a glass capillary, a hollow cantilever was employed to place single cells in microwells [17,18]. These two methods essentially have the same components.

Optical observation is a common method to obtain the locations and motions of objects. It is possible to automatically detect the capture of a single cell under microscope observation [14,16,19,20]. The capture of a cell can be determined by the cessation of the relative motion between the cell and a holder. Time-series microscope images contain 4D information (horizontal and vertical pixels, gray scale intensity, and time), which is often excessive. It is costly and complicated to process time-series microscope images. To help with the detection of a single cell, a different signal with a higher signal-to-noise (S/N) ratio is preferable. It is effective to use a time-series ionic current through a hollow channel, which provides 2D information containing the current and time. This current is more sensitive to the state of a tip opening. Therefore, the S/N ratio of the ionic current is higher than that of the optical method. A current has been used for measuring the electrophysiological activities of membrane channels, which is called the ‘patch-clamp technique’ [21,22], and for determining a surface topography, which is called ‘scanning ion-conductance microscopy’ [23,24,25,26]. Current measurement was applied to understand the insertion state of a needle in a single cell [27,28,29]. However, this current measurement method has never been extended to the field of single cell manipulation before. Establishing optimum conditions for achieving less-invasive and high release rates of an electrical measurement and pick-up and release method is desired. The application of the electrical detection method is expected to improve the robustness of cell manipulation.

In this work, we developed a pick-up and placement method for a single cell that is applicable to an open-top microwell plate using optical and electrical measurements. We used an ionic current through a pipette to detect the state of a cell on the pipette tip. A pipette and substrate were coated with agarose or proteins to improve the manipulation efficiency. We characterized the release rate from the pipette to reduce cell fouling and improve the efficiency of cell manipulation and we checked the cell membrane integrity to keep cells alive during manipulation with trypan blue. We found the conditions that are suitable for single cell manipulation and electrical detection. An applied voltage was adjusted to prevent the electroporation of a cell and the trace of an ejected cell was measured to place a cell at a target site.

## 2. Experimental Methods

### 2.1. Main Setup of Cell Manipulation and Measurement

**Figure 1:**
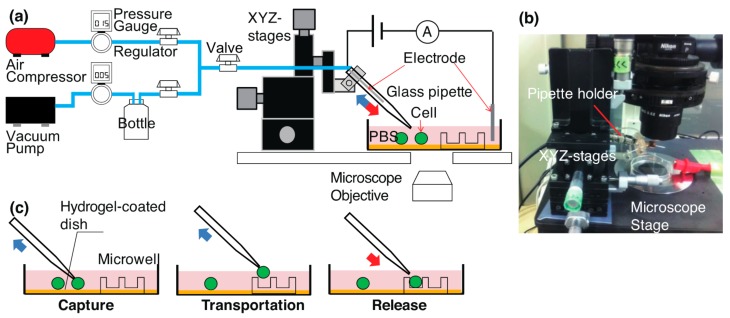
Experimental setup for cell manipulation and observation: (**a**) schematic and (**b**) image of setup, (**c**) schematic procedures for cell pick-up and placement in a microwell.

The experimental setup for cell manipulation and measurement is shown in Figure 1. A cell suspension was stored in a petri dish, which was covered with agarose gel to prevent the adhesion of cells. A glass pipette was connected to an electrode and pneumatic equipment through a pipette holder. The pressure on the pipette tip was controlled to capture and release a single cell. We used a custom-built pneumatic system and adjusted the pressure of the pipette to positive and negative pressures [30].

Figure 1c shows the cell pick-up and placement processes. A pipette was fixed on XYZ mechanical stages to move it. These stages were mounted on the sample stage of an inverted microscope. The pipette tip was moved close to a target cell to capture it, with the suction pressure set to −1, −3, or −5 kPa (gauge pressure). The cell was held at the tip and transported to a target microwell. The cell was released from the pipette tip by switching to a positive pressure of +5 or +15 kPa.

### 2.2. Cell Preparation and Membrane Integrity Assay

HeLa cells (RCB0007, derived from human cervical cancer cells) were purchased from Riken Cell Bank, (Tsukuba, Japan) and used to test the cell manipulation. These HeLa cells were cultured in a polystyrene dish (φ = 90 mm), which was placed in a multi-gas incubator (MCO-5M, Sanyo, Osaka, Japan). The incubator was maintained at 37 °C, 5% CO_2_, and 100% relative humidity. We used minimum essential medium (MEM 11095-080, Gibco, Thermo Fisher Scientific, Waltham, MA, USA) supplemented with 10% fetal bovine serum (FBS 171012, Nichirei Bioscience, Tokyo, Japan) as the culture medium. A cell suspension was obtained using the following process. The medium was removed from the dish, and the cells were rinsed with 5 mL of phosphate buffered saline (PBS 10010-123, Gibco, Thermo Fisher Scientific, Waltham, MA, USA). Then, 3 mL of 0.25% trypsin-EDTA (25200-072, Gibco, Thermo Fisher Scientific, Waltham, MA, USA) solution was added, they were then kept in the incubator for 5 min to detach the cells from the dish, and 3 mL of the medium was added to deactivate the trypsin. The solution was transferred to a 15 mL tube and centrifuged with a centrifuge machine (LC-220, Tomy Digital Biology, Tokyo, Japan) at 270 g for 3 min to increase the cell concentration. The supernatant containing trypsin was discarded, and 6 mL of phosphate buffered saline (PBS) was added to the tube.

We evaluated the effect of the pneumatic cell manipulation and electrical detection on the cell membrane integrity. To evaluate the cell membrane damage, we added trypan blue to the cell suspension. While trypan blue is not absorbed in viable cells, cells are stained when their membranes are damaged. The concentration of trypan blue in the cell suspension was adjusted to 0.2 *w*/*v* %. The cell membrane integrity was observed during and just after cell release.

### 2.3. Non-Adhesive Glass Pipette

We used a sharpened glass pipette to manipulate a single cell. The target I.D. for the pipette was 3–4 µm. We found that this diameter was suitable for cell manipulation [30]. A pipette puller (PC-10, Narishige, Tokyo, Japan) was used to make a glass pipette from a glass tube (I.D. 0.6 mm, O.D. 1.0 mm, GD-1, Narishige, Tokyo, Japan). We used four series of weights and two pulling steps with setting values of 70 at heater no. 1 and 60 at no. 2. The length of pulling was 5 mm for the first step and 2 mm for the second step.

To prevent unwanted cell adhesion, a glass pipette was coated with bovine serum albumin (BSA, B4287-5G, Sigma, St. Louis, MO, USA). The bovine serum albumin (BSA) solution was adjusted to 10 mg/mL in the PBS solution. The tip of the glass pipette was immersed in the solution and kept for 15 min at room temperature. The glass pipette was first washed with PBS and then filled with PBS. The coated pipette was used to place a single cell in a microwell. In the control experiment, the pipette was not coated with BSA.

### 2.4. Polydimethylsiloxane Microwell on Non-Adhesive Petri Dish

Cell fouling to a surface can interfere with cell manipulation. Therefore, we used a hydrophilic gel to prevent cells from adhering to the substrate [31]. We coated a polystyrene dish (50 mm in diameter) with agarose gel. Agarose powder (A9539-10G, Sigma, St. Louis, MO, USA) was dissolved in either PBS or 0.9% NaCl and adjusted to 2 wt %. The mixture was autoclaved at 121 °C for 20 min to fully dissolve the agarose powder. The agarose gel solution was kept at 80 °C and poured into a petri dish maintained at 60 °C on a hot plate. The gel solution was cooled in a refrigerator for 5 min to cure it. Before use, PBS was poured over the gel and kept for 5 min to saturate the gel with PBS.

We placed a polydimethylsiloxane (PDMS) microwell on a gel-coated dish and used it for the cell placement. The well was fabricated using a photolithography and PDMS molding process and each well had a diameter of 50 μm and depth of 30 μm. A silicon wafer was cleaned in a 3:1 (by volume) H_2_SO_4_ (96 wt %):H_2_O_2_ (30 wt %) mixture at 80 °C for 10 min. SU-8 3050 (Kayaku Microchem, Tokyo, Japan) was spin-coated on the wafer at 500 rpm for 25 s and 3000 rpm for 55 s. The wafer was baked at 65 °C for 5 min, 95 °C for 25 min, and 65 °C for 5 min. A mask aligner (PEM-800, Union Optical Co., Tokyo, Japan) was used to illuminate it with ultraviolet light through a microwell pattern until the light integral reached 300 mJ/cm^2^. The wafer was baked at 65 °C for 9 min, 95 °C for 5 min, and 65 °C for 2 min. The substrate was developed in 2-acetoxy-1-methoxypropane (Wako Chemical, Osaka, Japan) and rinsed with isopropyl alcohol (IPA).

PDMS (Silpot 184, Dow Corning Toray Co., Tokyo, Japan) was mixed at a 10:1 ratio of base polymer and curing agent by weight. An approximately 2 mm thick layer of uncured PDMS was poured over the SU-8 mold. The PDMS was baked at 80 °C for 60 min. The microwell was peeled off from the SU-8 mold and cut into pieces. To carry out cell capture and placement in the same dish, the microwell chip was placed at the center of the dish and the side of the PDMS chip was covered with agarose gel to fix the well chip.

### 2.5. Optical and Electrical Measurements During Cell Manipulation

The PBS and cell suspension were dispensed in a PDMS microwell and on the agarose gel, respectively. The cell suspension reduced the number of placed cells in the wells moved by a flow. A cell was captured on the agarose gel surface, where cell adhesion was weak. The captured cell was transported to a target microwell and ejected to the well.

The process of cell manipulation was recorded under an inverted microscope (ECLIPSE TE2000-U, Nikon, Tokyo, Japan) equipped with a charge-coupled device (CCD) camera (DIGITAL SIGHT DS-2Mv, Nikon, Tokyo, Japan). We measured the cell staining during the cell manipulation. We used the ionic current change through a pipette tip when a cell was trapped and released. The principle of electrical detection is to measure the current drop through a pipette when a cell increases the resistance. This current drop is caused by the sealing of the tip by the cellular membrane. Ag/AgCl wire electrodes (φ = 0.3 mm) were placed inside a glass pipette (cathode) and cell suspension (anode). A direct current (DC) power supply was used to apply a DC voltage between the electrodes and the current through the pipette was measured using a current source meter (428-PROG, Keithley, Cleveland, OH, USA) at intervals of 0.2 s. The amplitude of the current meter was 10^7^ V/A and was recorded with a data logger. The recorded currents were analyzed to understand the trend of the ionic current.

## 3. Results and Discussion

### 3.1. Electrical Detection of Single Cell

We measured the opening state of a single pipette by measuring the decrease in an ionic current.

**Figure 2:**
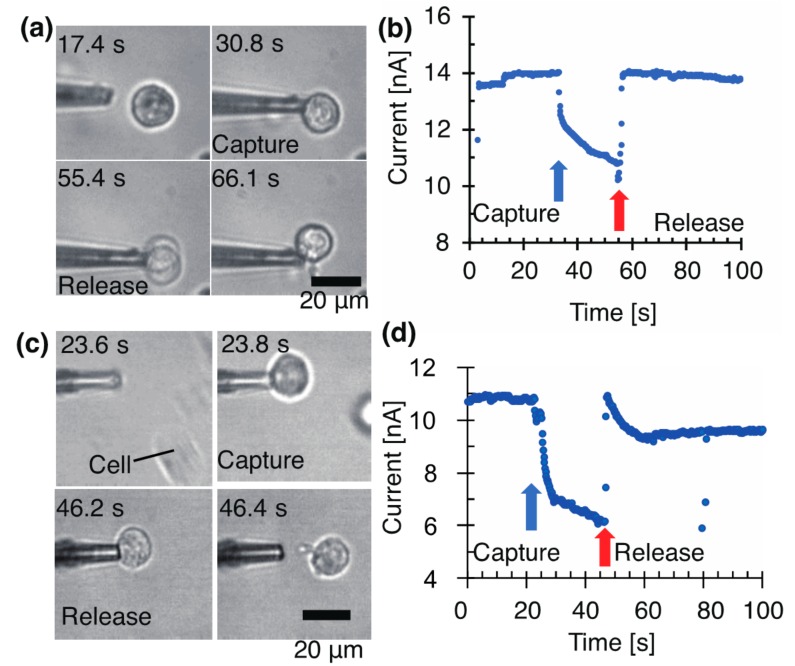
Manipulation and electrical detection of single cell with glass pipette at −5 and −1 kPa. The applied voltage was 100 mV. (**a**) Micrographs and (**b**) a current at −5 kPa with a pipette (I.D. 3.28 μm). (**c**) Micrographs and (**d**) a current at −1 kPa with a pipette (I.D. 3.26 μm).

Figure 2 shows the change in the current at 100 mV (DC) when a cell is trapped at the tip. The electrical change matched the optical observation of the cell trapping.

Figure 2a shows a current decrease of 1 nA in 0.4 s at −5 kPa caused by the increase in the tip resistance. The current drop was significantly different from the initial value. While the cell continued to be sucked, the current gradually decreased. At 23 s, the current decreased by 3 nA, which was caused by the increase in the resistance and the progress of the cell sealing. After the cell ejection at +15 kPa, the sealing of the pipette tip was cleared and the current returned to the initial value. The cell attached to the pipette and was not removed from the pipette. In this experiment, we did not coat the pipette with BSA to characterize its coating effect. The release of a cell was difficult to achieve, and 10/10 cells were not stained with trypan blue 200 s after cell capture at a suction pressure of −5 kPa.

Figure 2b presents a current decrease of 1 nA in 2.6 s at −1 kPa. Here, 25 s after the capture, the current decreased by 4.6 nA. The ejection at +5 kPa returned the current close to the initial value. It was 1–2 nA lower than the initial value of 11 nA. We consider this decrease to have been caused by the cell remaining on the opening. At −1 kPa, 8/8 cells were not stained with trypan blue for the observation time of 100 s.

In this experiment, the cells were suspended on a bare coverslip. Over time, the cells started to adhere to the surface and interfered with cell manipulation. The following sections discuss how cells were floated on a gel-coated coverslip, and the results are compared with the results outlined in this section.

### 3.2. Voltage Effect on Cell Membrane Integrity

**Figure 3:**
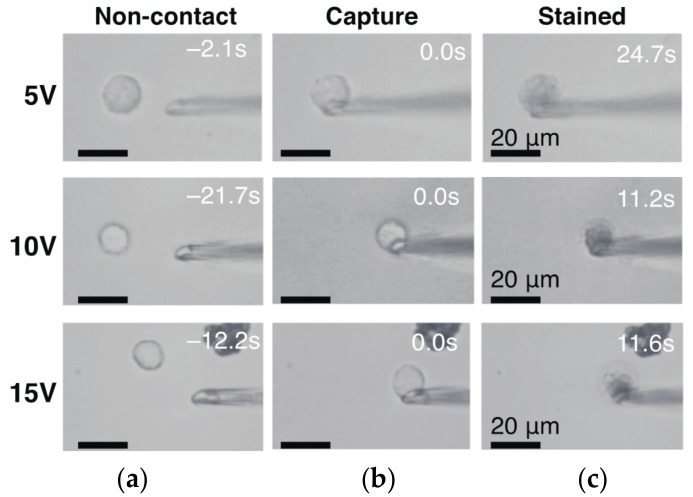
(black and white) Cell membrane integrity assay during cell manipulation and detection with pipette (I.D. 4.5 µm) at −5 kPa. (**a**) Cells before trapping. (**b**) Captured cells at the pipette tip. (**c**) Cells stained with trypan blue.

We set a DC applied voltage to +5, 10, and 15 V in a higher range and manipulated a cell at a constant suction pressure of −5 kPa. The major motivation of using higher voltage was to investigate the possibility in removal of a high-gain amplifier and simplification of the electrical measurement. The damage to the cell membrane was evaluated by staining the cell with a trypan blue solution (Figure 3). We used pipettes with I.D. of 3.3 µm and 4.5 µm. At 5 V_DC_, 6/7 and 1/7 cells were stained within 25 s and at 83 s from cell capture, respectively. At 10 V, 5/5 cells were stained within 15 s. At 15 V, 2/2 cells were stained within 12 s. The total number of intact cells was 0/14 (=0%). Although the time of the cell staining was the longest at +5 V among the three voltage conditions, 25 s or tens of seconds at 5 V was not sufficiently long for cell manipulation.

At an applied voltage of 100 mV, the cell viability increased as described in Section 3.1. Intact cells were 10/10 at −5 kPa and 8/8 at −1 kPa. Compared to the other single-cell manipulation methods, Z. Lu et al., reported 95% of cell viability using a glass micropipette [16]. By contrast to 100 mV, the number of intact cells was 0/14 at values greater than 5 V. Decreasing the voltage was useful to increase the viability. This voltage effect on cell viability can be explained by the fact that a cell membrane has a breakage voltage threshold of 150–500 mV [32]. The aspirated portion of the membrane is thought to be porated with a high electric field.

### 3.3. Increasing Cell Release by BSA Coating and Motion Behavior of Cell Released from Pipette Tip

**Figure 4:**
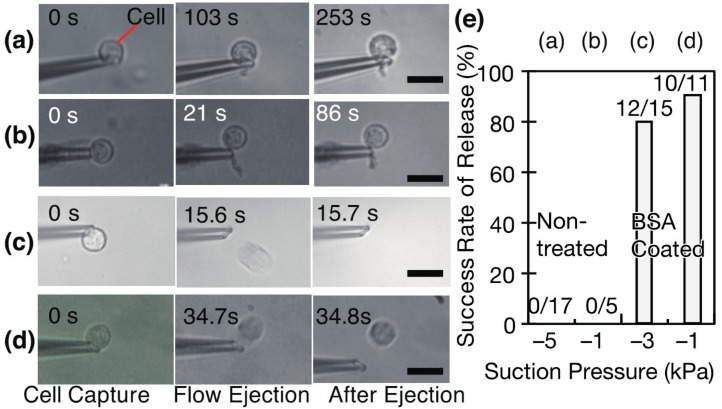
Release rates of cells from tips of (**a**,**b**) non-coated and (**c**,**d**) BSA coated pipettes. The concentration of cells was 2.94 × 105 cells/mL. Measurement results of cell release after its capture on the tip (**e**).The experimental conditions for (**a**–**d**) are listed in Table 1.

A pipette was coated with BSA and the coating enhanced the release of a cell from the tip (Figure 4 and Table 1). The cell remained at the pipette (Figure 4a,b, Video S1) after ejection. On the contrary, the cell was fully removed from the pipette as shown in Figure 4c,d. The cell floated on an agarose gel and was captured on the tip by suction. With a BSA-coated pipette, the total release rates within 20 s were 80.0% (=12/15) at −3 kPa and 90.9% (10/11) at −1 kPa, with 9/15 cells released immediately after ejection at −3 kPa. Some part of 3/15 cells adhered to the tip and released in 20 s, whereas 2/15 cells remained 70 s after the start of the ejection. We checked that the effect of BSA coating continued for at least 30 min and five times cell capture and release.

**Table:** 

Legend	BSA Coating	Suction Pressure	Ejection Pressure	Pipette I.D.
(a)	Non-coat	−5 kPa	+15 kPa	2.86, 3.22, 3.28 μm
(b)		−1 kPa	+5 kPa	3.20 μm
(c)	Coat	−3 kPa	+5 kPa	2.79, 2.91 μm
(d)		−1 kPa	+5 kPa	2.78, 3.03 μm

Without the BSA coating, the cells were not released from the pipette tips. The release rates were 0/17 at −5 kPa and 0/5 at −1 kPa. We concluded that the increase in the release rate was from the effect of the BSA treatment and that the BSA coating significantly improved the cell release.

**Figure 5:**
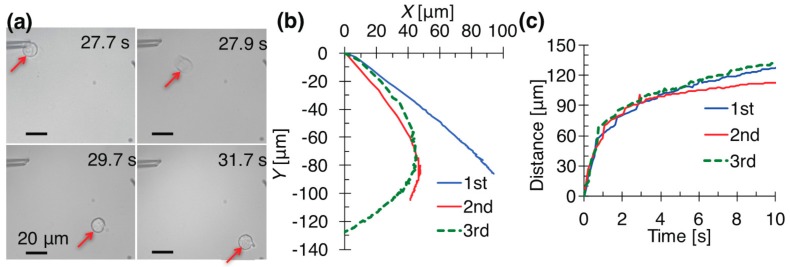
Ejection of three single cells from pipette (I.D. 2.91 μm) at +5 kPa. (**a**) Time-lapse micrographs of the first manipulated cell; (**b**) trajectory of the single cells; (**c**) distances of the cells from the pipette tip.

The trace of a cell ejected from a pipette (Video S2) was measured to reduce the placement error of a cell in a microwell (Figure 5). An ejection at +5 kPa generated a continuous flow from a tip and transported a cell along the flow. The travel direction of the cell was affected by the opening of the tip and a flow disturbance. The origin of the *X*–*Y* coordinates was set at the tip of the pipette. We measured the distance of the cell from the origin. The same pipette moved single cells to the lower right (Figure 5b). The angle of the opening affected the cellular motion, because the flow and pressure were generated according to the direction of the opening. The third cell in Figure 5 changed its direction to the lower left. A flow disturbance to the left was generated.

The distance that a cell moved from the tip versus the time is plotted in Figure 5c. This graph shows that the flow speed decreased over time. The pipette flow became weak at a large distance from the tip. A cell ejected at +5 kPa stopped at a distance of approximately 140 μm from the pipette. To place a cell at a target location, it was necessary to consider the following things: (1) a microwell to reduce the flow and maintain the cell; (2) the direction of the tip opening; (3) the travel distance from the tip; (4) any flow disturbance. Among these things, a microwell is the key component to place a cell in a position with high precision.

### 3.4. Placement of Single Cells in Microwell

**Figure 6:**
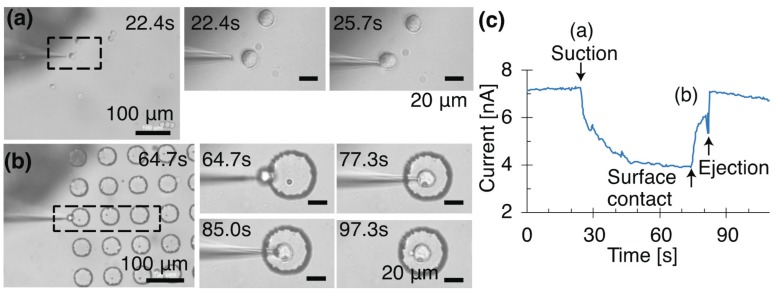
Time-lapse micrographs of first cell transported into microwell. The cell was captured at −3 kPa with an applied direct current (DC) voltage of 100 mV. The cell ejected from the pipette (I.D. 2.91 μm) at +5 kPa. (**a**) Cell capture on an agarose gel; (**b**) cell release in a microwell; (**c**) current through a pipette tip during cell manipulation.

We placed a single cell in a target microwell using optical and electrical observations. A cell was captured at −3 kPa on the agarose gel (Figure 6, Video S3) with a glass pipette (I.D. 2.79 μm) treated with BSA. HeLa cells were suspended in PBS, and the concentration was adjusted to 1.36 × 10^5^ cells/mL. The recorded microscope video and current provided visual and electrical information, respectively. The manipulation was observed under optical microscopy, and the current through the pipette tip was recorded. The capture of a single cell decreased the ionic current (Figure 6c). The cell was held at the tip and transported to a microwell with a diameter of 50 μm. When approaching the well, the cell touched the surface of the well (Figure 6b). After contact with the surface, the current increased, because the cell moved from the tip and the tip seal was broken. Because of the softness of the PDMS microwell and the flexibility of the glass pipette, we did not observe breakage of the pipette. When the cell was ejected at +5 kPa, it was released from the pipette. After its release, the cell remained in the well. Gentle contact did not cause the deformation of the cell.

**Figure 7:**
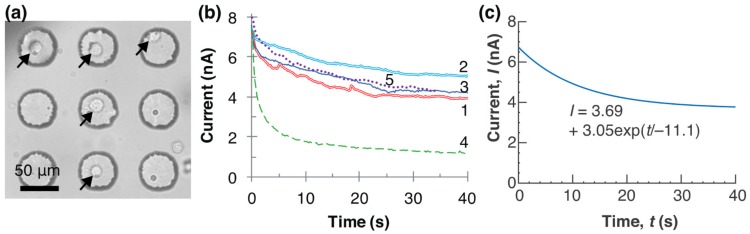
Formation of letter ‘T’ by picking up and placing five cells in microwells using pipette. (**a**) Micrograph of the five placed cells; (**b**) five current curves versus time from cell capture. Current no. 1 is also shown in Figure 6; (**c**) averaged curve of the five fitted curves using I=A+Bexp(t/C). *A* = 3.69 ± 1.30 (nA), *B* = 3.05 ± 1.05 (nA), and *C* = −11.1 ± 5.47 (s) (*n* = 5).

After the placement of the first cell, the pick-up and placement process was repeated four times. Four more cells were placed to make a T-shaped pattern using a total of five cells on the microwell chip (Figure 7). Figure 7a shows the five cells placed inside the microwells. After the flow ejection, some of the cells remained on the pipette. Touching them on the well edge released the cells inside the microwell. For cells adhering to the pipette, physical contact was effective at releasing them from the pipette.

Five series of currents were recorded as shown in Figure 7b. Since the aspirated length of a cell can be expressed as an exponential function of time [33], we assumed that the aspirated portion of a cell increased the resistance of the circuit. The five currents from the cell capture were fitted to exponential decay curves given by the equation

(1) I=A+Bexp(t/C)

Parameters *A*, *B*, and *C* were estimated using the least squares method. Figure 7c is the summarized curve of the ionic currents after capturing the cells. The three parameters were averaged. The relationship between the current through the pipette (nA), *I,* and time (s), *t,* is described by Equation (2).

(2) I=3.69+3.05exp(t/−11.1)

The results of the electrical detection indicated that the current exponentially decayed with time. The results were well fitted to the equation and this fact supports our assumption. The measured current profiles had the same trends and could detect the cell capture and release. The electrical measurement was an effective and easy method of processing a single cell detection.

This method provided the 2D (current *I* and time *t*) qualitative information at 0D (point). This current information was more sensitive to the contact between a cell and pipette than the 4D optical information that is 2D information (gray-scale intensity *I_g_*, and time *t*) on a 2D area (horizontal size *p_x_*, vertical size *p_y_*) [14,19,20]. *p_x_*, *p_y_* of typical images are in the order of 100 pixels. These values give us an interrogation area of 2D images in the order of 10^4^ pixel, which is far wider than 0D current information. This fact means that the S/N ratio of the ionic current is higher than that of the optical method.

We could apply this detection method to an array of micro-nozzles made by microfabrication [30,34,35] in order to pick-up and place single cells in a parallel manner. Microelectrodes in the nozzle array and multi-channel amplifiers [36] allow for measurement of the ion current through each nozzle at the same time. A device with pneumatic valves [37,38] would allow for individual control of the capture and release processes. An array of nozzles could approach a target substrate where negative pressure could be used to trap single cells on the nozzles, after which they could be placed at target sites.

## 4. Conclusions

We developed a pick-up and placement method for single cells that is compatible with open top microwells using optical observation and electrical detection for robust cell manipulation. The electrical current was sufficiently sensitive to detect a single cell with the reduced 2D information. The use of image processing and current measurement could realize a more robust screening method for single cells.

We found the conditions suitable for single cell manipulation and electrical detection. Decreasing the voltage was effective at keeping the cells alive during cell manipulation. The state of a single cell on a glass pipette was measured using a current through the pipette. A voltage of 100 mV was sufficiently low to keep the cells alive.

A glass pipette coated with BSA was useful for releasing the cells from the pipette. This coating was essential to achieve cell manipulation. After the BSA-coating, the rate of cell release increased. The movement of a cell was characterized to reduce its movement. The flow ejected from the pipette affected the motion of the cell and we found that microwells reduced the movement after the release. Repeated placements in microwells were demonstrated, and five single cells were placed in target microwells. We developed a repeatable method of single cell manipulation with the help of optical observation and electrical detection. A current constantly decreased during cell trapping and increased after cell release. The detection of this trend can assure successful cell displacement procedures. Our methods have the potential to be extended to a method for the automated pick-up and placement of single cells.

## Figures and Tables

**Table 1:** Experimental conditions for characterization of cell release described in Figure 4

Legend	BSA Coating	Suction Pressure	Ejection Pressure	Pipette I.D.
(a)	Non-coat	−5 kPa	+15 kPa	2.86, 3.22, 3.28 μm
(b)		−1 kPa	+5 kPa	3.20 μm
(c)	Coat	−3 kPa	+5 kPa	2.79, 2.91 μm
(d)		−1 kPa	+5 kPa	2.78, 3.03 μm

## Supplementary Materials

The following videos are available online at www.mdpi.com/2072-666X/8/12/350/s1; Video S1: Cell release with non-coated pipettes at a pressure of 15 kPa; Video S2: Time-sequence of the first cell manipulation (two times faster than real time); Video S3: picking up and placing a single cell in a microwell using a pipette (two times faster than real time).
